# Deciphering the Contributions of CRH Receptors in the Brain and Pituitary to Stress-Induced Inhibition of the Reproductive Axis

**DOI:** 10.3389/fnmol.2018.00305

**Published:** 2018-08-30

**Authors:** Androniki Raftogianni, Lena C. Roth, Diego García-González, Thorsten Bus, Claudia Kühne, Hannah Monyer, Daniel J. Spergel, Jan M. Deussing, Valery Grinevich

**Affiliations:** ^1^Schaller Group on Neuropeptides, German Cancer Research Center, Heidelberg – Central Institute of Mental Health, Mannheim, Germany; ^2^Department of Molecular Neurobiology, Max Planck Institute for Medical Research, Heidelberg, Germany; ^3^Department of Clinical Neurobiology, Medical Faculty of Heidelberg, University of Heidelberg – German Cancer Research Center, Heidelberg, Germany; ^4^Max Planck Research Group at the Institute for Anatomy and Cell Biology, University of Heidelberg, Heidelberg, Germany; ^5^Molecular Neurogenetics Research Group, Department of Stress Neurobiology and Neurogenetics, Max Planck Institute of Psychiatry, Munich, Germany; ^6^Department of Neurosurgery, Yale University School of Medicine, New Haven, CT, United States

**Keywords:** stress, reproduction, CRH, CRH receptors, GnRH, GnRH neurons, gonadotropes

## Abstract

Based on pharmacological studies, corticotropin-releasing hormone (CRH) and its receptors play a leading role in the inhibition of the hypothalamic–pituitary–gonadal (HPG) axis during acute stress. To further study the effects of CRH receptor signaling on the HPG axis, we generated and/or employed male mice lacking CRH receptor type 1 (CRHR1) or type 2 (CRHR2) in gonadotropin-releasing hormone neurons, GABAergic neurons, or in all central neurons and glia. The deletion of CRHRs revealed a preserved decrease of plasma luteinizing hormone (LH) in response to either psychophysical or immunological stress. However, under basal conditions, central infusion of CRH into mice lacking CRHR1 in all central neurons and glia, or application of CRH to pituitary cultures from mice lacking CRHR2, failed to suppress LH release, unlike in controls. Our results, taken together with those of the earlier pharmacological studies, suggest that inhibition of the male HPG axis during acute stress is mediated by other factors along with CRH, and that CRH suppresses the HPG axis at the central and pituitary levels via CRHR1 and CRHR2, respectively.

## Introduction

It is well accepted that stress has a negative impact on reproductive function (Lovejoy and Barsyte, 2011). An initial speculation that stress suppresses reproduction was based on the early observations of Selye (1939). Fifty years later, it was demonstrated that the central neuropeptide of the hypothalamic–pituitary–adrenocortical (HPA) axis, corticotropin-releasing hormone (CRH), profoundly suppresses the activity of the hypothalamic–pituitary–gonadal (HPG) axis mainly at the central level (Rivier and Vale, 1984; Rivier et al., 1986; Petraglia et al., 1987; Chrousos and Gold, 1992; Kalantaridou et al., 2010; Kageyama, 2013). Studies also showed that central infusion of CRH receptor antagonists reverses acute stress-induced inhibition of luteinizing hormone (LH) levels in rats (Rivier et al., 1986; Maeda et al., 1994; Rivest and Rivier, 1995), suggesting that CRH receptor signaling is required for stress-induced suppression of the HPG axis.

Gonadotropin-releasing hormone (GnRH) is the central hormone of the HPG axis, acting via LH and follicle-stimulating hormone (FSH) in the pituitary to trigger the synthesis and release of sex steroids and promote gametogenesis by the gonads (Amoss et al., 1971; Schally et al., 1971a,b; Seeburg et al., 1987). GnRH neurons are scattered throughout the basal forebrain. In rats and mice, GnRH neurons are most abundant in the medial septum (MS), diagonal band of Broca (DBB), preoptic area (POA), and anterior hypothalamic area (AHA) (Silverman et al., 1987; Jasoni et al., 2009), from where they project axons to the external zone of the median eminence and release GnRH in pulses: ∼1 pulse every 30 min (Katt et al., 1985; Czieselsky et al., 2016) based on measurements of LH. GnRH reaches the pituitary via the hypophyseal portal circulation (Levine and Ramirez, 1982), binds to GnRH receptors on gonadotropes, and stimulates the release of LH and FSH into the bloodstream (Gharib et al., 1990; Lopez et al., 1998; Bliss et al., 2010; Gongrich et al., 2016).

Corticotropin-releasing hormone is well known as the central hormone of the HPA axis, controlling neuroendocrine and homeostatic responses to stress (de Kloet et al., 2005). CRH acts on neurons and pituitary cells mainly via two types of G-protein coupled receptors, CRH receptor types 1 and 2 (De Souza, 1995; de Kloet et al., 2005) encoded by two different genes that are roughly 70% homologous in their amino acid sequence (Perrin and Vale, 1999). CRHR1 is expressed abundantly throughout the brain, whereas CRHR2 expression is restricted to a few brain regions but dominates in the periphery (Hauger et al., 2006). Moreover, CRH seems to act by primarily binding to CRHR1, since it has more than a 10-fold higher affinity for this receptor than for CRHR2 (Hauger et al., 2006). The role and involvement of CRHR1 and CRHR2 in controlling HPA axis activity, emotionality, and autonomic functions are extremely complex and not fully understood (Reul and Holsboer, 2002). In numerous studies, CRH acting via CRHR1 has been proposed as the main factor suppressing GnRH and LH release during acute stress (MacLusky et al., 1988; Dudas and Merchenthaler, 2002; Jasoni et al., 2005; Todman et al., 2005; Li et al., 2006), and to do so by acting directly and/or indirectly (particularly via GABAergic neurons) on GnRH neurons (Rivier and Vale, 1984; Nikolarakis et al., 1986; Petraglia et al., 1987; MacLusky et al., 1988; Cates et al., 2004; Li et al., 2010, Li et al., 2011; Lin et al., 2011).

Despite a plethora of studies, the interplay between the hormones of the HPA and HPG axes with respect to reproductive function as well as the precise site of action of CRH signaling within the HPG axis is not yet fully understood. Therefore, in the present study, by employing a variety of transgenic mice carrying deletions of CRH receptors in GnRH neurons, GABAergic neurons, or all central neurons and glia, we investigated the roles of CRHRs in the inhibition of HPG axis activity under psychological or immunological stress, which are considered to be two different types of acute stress (Grinevich et al., 2001a,b).

## Materials and Methods

### Animals

Male GnRH-GFP transgenic mice (**Table 1**) ranging in age from postnatal day (P) 28, which is around the time at which the HPG axis becomes active (Bell, 2018), to P44 were used for *ex vivo* electrophysiology experiments, whereas male transgenic (various transgenes; **Table 1**) and WT mice ranging in age from P90 to P180 were used for stress, infusion, and cell culture experiments. Mice were kept under standard laboratory conditions (12/12 h light/dark cycle, lights on at 07:00 h; room temperature 22 ± 2°C; 55 ± 5% relative humidity) with access to water and standard mouse chow *ad libitum*. All experiments were approved (animal protocol numbers, 35-9185.81/G-241/12, and its extension, 35-9185.81/G-314/14) by the ethics committee of the Regierungspräsidium Karlsruhe (Germany) and conducted in accordance with the European Communities Council Directive of 22 September 2010 (2010/68/EU).

**Table 1:** List of transgenic mouse lines/strains.

N	Line/Strain	Reference	Experiment	Figure	n
1	GnRH-CreERt2 (GnERt)	This paper	Basal	**Figure 1**	7
2	GnRH-CreERt2-Rosa	This paper	Brain histology	**Figure 1**	6
3	C57BL/6		Basal and TAM-treated	**Figure 1** and **Supplementary Figure S1**	33
4	CRHR1-2LoxP	Muller et al., 2003	Restraint, LPS, Basal, ASCF/CRH infusion	**Figures 4A1–B2, 5B1,B2**	12, 12, 16, 8/7
5	GnRH-CRHR1 CKO	This paper	Restraint, LPS, Basal, *In situ* hybridization	**Figure 2** and **Supplementary Figure S2**	12, 17, 13, 12
6	CRHR1-GFP	Justice et al., 2008	Histology	**Figure 3A**	3
7	GnRH-GFP	Spergel et al., 1999	Brain slice electrophysiology	**Figures 3B–E**	3
8	Dlx-CRHR1 CKO	This paper	Restraint, LPS injection	**Figures 4A1,A2**	5, 8
9	Nestin-CRHR1 CKO	Schmidt et al., 2000	Restraint, LPS injection, ACSF/CRH infusion	**Figures 4B1,B2, 5B1,B2**	12, 12, 7/7
10	Nestin-CRHR2 CKO	This paper	Restraint, LPS injection, ACSF/CRH infusion	**Figures 4C1,C2, 5C1,C2**	9, 8, 5/6
11	CRHR2-2LoxP	Henckens et al., 2017	Restraint, LPS injection, Basal, ACSF/CRH infusion	**Figures 4C1,C2, 5C1,C2**	9, 9, 6, 5/6
12	CRHR1-KO	Refojo et al., 2011	Pituitary cell culture	**Figures 6A1,A2**	4
13	CRHR2-KO	Coste et al., 2000	Pituitary cell culture	**Figures 6B1,B2**	10
14	CRHR1-Cre/Ai9	Dedic et al., 2018	Pituitaries/immunostaining	**Figure 7**	3
15	CRHR2-Cre/Ai9	Henckens et al., 2017	Brain histology, Pituitaries/immunostaining	**Supplementary Figure S4** and **Figure 7**	2
Total number of animals					324

### Generation and Characterization of GnRH-CreERt2 Mice

#### Generation of GnRH-CreERt2 Mice

**FIGURE 1:**
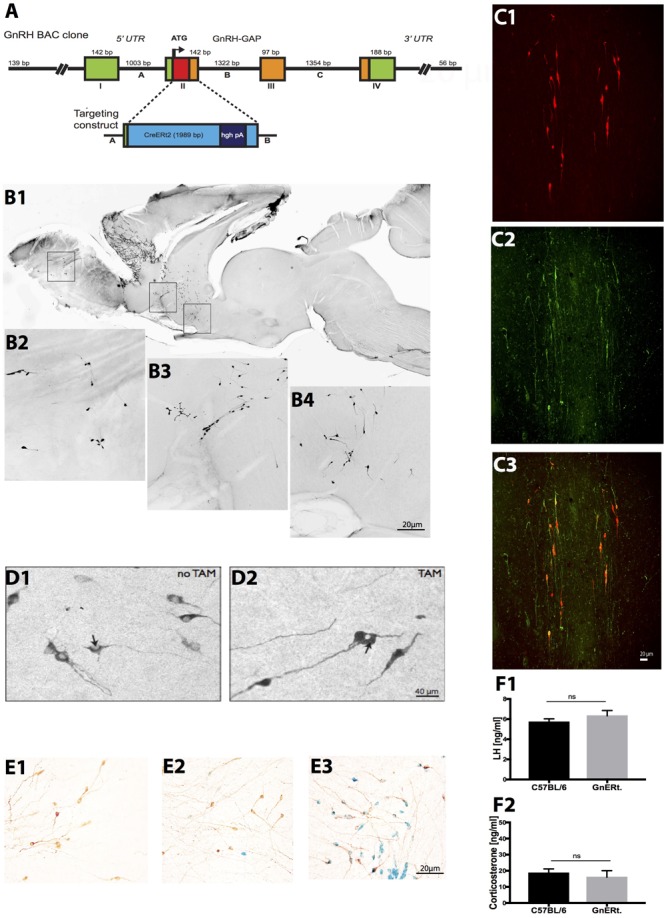
Generation and characterization of GnRH-CreERt2 BAC mice. **(A)** Schema of the GnRH-CreERt2 targeting vector. Bacterial artificial chromosome (BAC) containing the GnRH promoter driving the expression of the fusion proteins of modified estrogen receptor alpha ligand binding protein and improved Cre recombinase (CreERt2). The four exons (I–IV) of the GnRH gene are presented as boxes, the three introns (A–C) as lines in between. GAP, GnRH-associated peptide; hgh pA, polyadenylation sequence of the human growth hormone; UTR, untranslated region. **(B)** Immunocytochemical analysis of a sagittal brain slice from a GnRH-CreERt2 mouse **(B1)** shows that Cre expression resembles the pattern of GnRH expression in the olfactory bulb **(B2)**, terminal nerve **(B3)**, and preoptic area **(B4)**. **(C)** Colocalization of Cre and GnRH. Coronal sections of GnRH-CreERt2 mouse brain were stained for Cre (visualized using Cy3, red; **C1**) and GnRH (visualized using FITC, green; **C2**). The overlay of both channels shows that virtually all GnRH neurons express Cre **(C3)**. **(D)** Tamoxifen-dependent translocation of Cre recombinase from cytoplasm to nucleus. Sections were stained for CreERt2 (visualized using DAB). In untreated GnRH-CreERt2 mice, Cre resides in the cytoplasm **(D1)**, while injection of tamoxifen induces translocation of Cre into the nucleus (**D2**, indicated by arrow). **(E)** Tamoxifen-induced Cre recombination in GnRH neurons, assessed using combined X-gal staining for β-galactosidase activity and immunostaining for GnRH. Vehicle (oil) injection did not induce β-galactosidase activity in GnRH neurons (**E1**, brown color). A single tamoxifen injection resulted in the appearance of β-galactosidase activity (blue color) in single GnRH neurons **(E2)**. Ten injections of TAM induced β-galactosidase activity exclusively in GnRH neurons, and in virtually all GnRH neurons **(E3)**. **(F)** Plasma concentrations of LH **(F1)** and corticosterone (CS; **F2**). LH and CS concentrations did not differ between C57BL/6 WT (*n* = 14) and GnRH-CreERt2 (GnERt) mice (*n* = 5). Results are expressed as mean ± SEM; unpaired *t*-test, *p* > 0.05; ns, no significant statistical difference.

In the present study, we used 14 transgenic mouse lines, most of which were generated and/or characterized in our labs previously (**Table 1**). To generate the GnRH-CreERt2 mouse line, which has not been described previously, a 208 kb BAC containing the GnRH locus (∼140 kb upstream and ∼50 kb downstream of the ATG start codon of the GnRH gene), which corresponds to clone 12B1 on mouse chromosome 14 (Korenberg et al., 1999), was isolated from a 129 SV mouse BAC library (cat. no. 96022, Thermo Fisher Scientific, Menzel GmbH, Braunschweig, Germany). The targeting construct ERt2iCre.FrtNeoRFrt (CreERt2) for homologous recombination encodes the fusion protein of modified estrogen receptor alpha ligand binding protein and a codon-improved Cre recombinase (T. Wintermantel, DKFZ, Heidelberg) followed by the polyA signal of the human growth hormone gene (hgh pA) and the Frt-flanked neoR (Neomycin resistance as a selection marker) cassette (**Figure 1A**). The targeting construct was flanked with 60 nt homologous sequences upstream of the ATG (“arm” A) and downstream of exon II of the GnRH (“arm” B; GenBank no. M14872) by polymerase chain reaction (PCR) amplification with overhang primers. The 214 kb BAC containing the GnRH locus was recombined in *E. coli* EL250 cells (kindly provided by Dr. Neal Copeland, Mouse Cancer Genetics Program, Center for Cancer Research, National Cancer Institute, Frederick, MD, United States), as previously described (Lee et al., 2001). After removal of the selection marker, the modified BAC DNA was purified on Sepharose CL-4B columns and microinjected into pronuclei of B6/CBAF1 oocytes (S. Dlugosz and F. Zimmermann, IBF, Heidelberg, Germany). Eleven transgenic founders were identified by tail PCR analysis and backcrossed with C57BL/6 mice. Nine founders transmitted the gene to the offspring. All founder lines were analyzed as described in the section “Results”, and one line (line # 9) was selected, based on its specificity and inducibility of Cre expression, for experiments.

#### Characterization of Specificity and Recombination Efficiency of GnRH-CreERt2 Mice

To characterize the specificity of Cre expression, all GnRH-CreERt2 founder lines were analyzed. Immunohistochemistry revealed that the Cre expression matched the pattern of GnRH expression (**Figure 1B**). Double immunofluorescence staining for GnRH and Cre showed that virtually all GnRH neurons express Cre (**Figure 1C**). Furthermore, without tamoxifen (TAM) treatment Cre remained in the cytoplasm, but was translocated into nuclei after TAM injection (**Figure 1D**). This demonstrates that CreERt2 cannot be activated by endogenous estrogens and therefore allows a time-specific recombination of the floxed gene. Next, GnRH-CreERt2 mice carrying a copy of the gene-targeted Rosa26 floxed-stop lacZ locus (Soriano, 1999) were generated to verify the sufficiency of Cre-catalyzed recombination of the Rosa26 locus. Animals were treated either with a single TAM intraperitonially (i.p.) injection (1 mg) or with 10 i.p. injections over five consecutive days (two injections per day, 1 mg per injection). Control animals were injected with vehicle (oil) only. Combined X-gal staining for β-galactosidase activity and immunostaining using (DAB) for GnRH revealed that 10 i.p. injections over five consecutive days sufficiently induced recombination of the Rosa locus (**Figure 1E**), whereas vehicle or a single TAM injection did not induce efficient recombination.

Prior to performing stress experiments, we verified that basal plasma LH and corticosterone (CS) concentrations in GnRH-CreERt2 (also referred to as GnERt) mice were similar to those of wild type (WT) littermates (**Figures 1F1,F2**).

#### Tamoxifen (TAM) Treatment of GnRH-CreERt2 Mice

To induce Cre recombination, GnRH-CRHR1-CKO mice were injected i.p. with 1 mg of tamoxifen (Sigma-Aldrich, St. Louis, MO, United States) dissolved in sunflower oil (Sigma-Aldrich) and EtOH (100%) two times a day for 5 days (stock solution: 50 mg TAM in 4.5 ml sunflower oil and 0.5 ml EtOH 100%). Four weeks after the last tamoxifen injection, one group of GnRH-CRHR1-CKO was used for stress experiments, while six mice were killed for double *in situ* hybridization (dISH) without subjecting them to stress. Additionally, for control purposes, we wanted to evaluate a putative effect of tamoxifen (TAM) under basal conditions, as administration of an estrogen receptor antagonist such as TAM may affect the activity of GnRH neurons and consequently plasma LH levels. For this purpose, C57BL/6 wild-type and GnRH-CRHR1 CKO mice were injected with TAM (as described above), gonadectomized 4 weeks after the last injection, and killed 1 week later to collect blood. Under basal conditions there was no statistically significant difference in LH and CS concentrations in C57BL/6 wild-type or GnRH-CRHR1 CKO mice treated with TAM compared to untreated C57BL/6 wild-type mice. This indicates that TAM as an estrogen-related compound does not affect basal concentrations of LH and CS (**Supplementary Figure S1**).

#### Gonadectomy

For *in vivo* experiments [stress experiments and intracerebroventricular (i.c.v.) CRH infusion experiments], we subjected male mice to gonadectomy (orchidectomy) to avoid negative feedback signaling of circulating sex steroids and to increase detectability of LH. A small incision directly above the scrotum was made under a mixture of ketamine hydrochloride (90 mg/kg) and xylazine (5 mg/kg) anesthesia dissolved in phosphate buffered saline (PBS). Both testes were extracted and the incision was ligated with a monofilament suture and removed. Muscle layers and skin were sutured, and mice were singly housed until full recovery from the anesthesia. One week after surgery, stress experiments were conducted and blood was collected for measurement of plasma LH and CS levels. For the non-stressed control groups, blood was collected without subjecting mice to stress.

### Stress Experiments

Stress experiments were performed 1 week after gonadectomy. Mice were group-housed until the day before the experiment. The evening before the experiment, mice were brought into the experimental room to habituate to the environment. Stress experiments were performed for 1 h between 8 am and 11 am. For immobilization stress (restraint), mice were immobilized in a 50-ml falcon tube (11.5 cm long, 3 cm wide; Greiner Bio-One GmbH, Frickenhausen, Germany), which had a hole on one side to allow normal breathing. Remaining within the tube, mice were left undisturbed for 1 h in a cage. After 1 h of restraint, mice were immediately removed from the restraint device and killed, then blood was collected as described below. To model inflammatory stress, mice were injected i.p. (dose 2 μg/g b.w., concentration 0.2 mg/ml, L3012-5MG; Sigma-Aldrich) with the endotoxin LPS (Spinedi et al., 2002). After peripheral administration of LPS, mice were left undisturbed in their home cage. One hour later, animals were sacrificed and blood was taken. Mice were sacrificed using a brief overdose of CO_2_ (while isoflurane was used for euthanizing mice prior to *in situ* hybridization, and immunohistochemistry experiments), which has been shown not to affect HPA axis activity (Hackbarth et al., 2000; Grinevich et al., 2011; Valentine et al., 2012), and blood was obtained from the inferior vena cava. Blood samples were collected into a 1 ml syringe, containing 50 μl ethylenediaminetetraacetic acid (EDTA. 0.5 M, pH 8; Sigma-Aldrich), transferred into an EDTA coated tube (Becton Dickinson, Franklin Lakes, NJ, United States), and centrifuged within 20 min after withdrawal for 20 min at 2,000 rpm and 4°C. Plasma was stored at -80°C until radioimmunoassay (RIA) measurements of LH and CS.

### Intracerebroventricular Infusion of CRH

For i.c.v. drug infusion, guide cannulas were implanted stereotaxically 2 mm above the right lateral ventricle (LV) (ML +1, AP +0.2, DV -1.4). The stainless steel guide cannula (8 mm length, 26GA, Plastics One, Roanoke, VA, United States) was anchored to the skull with two screws (Plastics One) and dental cement (Paladur, Heraeus Kulzer, Hanau, Germany) and closed with a dummy (7 mm length, Plastics One). After recovery from surgery (implantation of cannula and gonadectomy), mice were singly housed (to prevent damage to the cannula) until the day of the experiment and handled twice a day for 5 days to minimize non-specific stress responses during the experiment. Mice received either an i.c.v. infusion of artificial cerebrospinal fluid (ACSF), which contained (in mM): 124 NaCl, 3 KCl, 1.8 MgSO4, 1.6 CaCl_2_, 1.25 NaH_2_PO_4_, 26 NaHCO3, and 10 glucose (pH 7.4), or of ACSF plus 200 μg human recombinant (h/r) CRH (Tocris, cat. no. 1151) (Radulovic et al., 1999). Each mouse was gently restrained by hand, the dummy was removed, and ACSF or 1 μl of h/r CRH (200 ng/μl) was injected through the guide cannula over a period of 1 min. The injection cannula was left in place for another 30 s to allow for drug diffusion. The injection cannula was made of stainless steel (10 mm length, 33GA, Plastics One) and connected to a 5 μl Hamilton microsyringe with polyethylene-50 tubing. The injection cannula extended 2 mm below the end of the guide cannula into the LV.

### Pituitary Cell Culture

Adult (3–6 months old) global CRHR1-KO, CRHR2-KO mice, and their respective wild-type littermates (for control purposes) were used. After decapitation under isoflurane anesthesia, the anterior pituitary glands were dissected out in ice-cold Hanks balanced salt solution (HBSS; Life Technologies) plus 10 mM HEPES. Following 45 min incubation at 37°C in 0.25% trypsin, cells were triturated in the presence of 0.05% DNAse I. Trypsin was blocked with 10% FBS. Cells were washed twice with DMEM (Life Technologies) without phenol red and plated in a poly-L-lysine (10 μg/ml in borate buffer, pH 8.4) coated 24 well plate at 8.7 × 10^4^ cells per well on average for the experiments using pituitaries from CRHR1-KO mice and their WT littermates and at 1.1 × 10^5^ cells per well on average for the experiments using pituitaries from CRHR2-KO and their WT littermates, respectively. DMEM containing FBS (5%), Pen/Strep (1%), non-essential amino acids (1%), sodium pyruvate (1%), and GlutaMAX (1%) (Life Technologies) was used as culture medium. After 3 days *in vitro* (DIV), the cells underwent a 6 h serum starvation and were then stimulated with 10^-8^ M CRH (Sigma-Aldrich, cat. no. C3042) for 6 h.

### Radioimmunoassay of LH, CS, or ACTH in Plasma and in Pituitary Cell Culture Medium

Mouse LH radioimmunoassay (RIA) was performed by the Endocrine Technology and Support Lab, Oregon National Primate Research Center (Beaverton, OR, United States) using a traditional double-antibody RIA procedure similar to that described previously (Pau et al., 1986). The LH RIA kit was purchased from Dr. Albert Parlow (NHPP, Harbor-UCLA Medical Center, Los Angeles, CA, United States) and included a vial of rat LH (NIDDK-rLH-I-8, a.k.a. AFP-12066B; about 100 μg) for iodination, a vial of mouse LH (AFP-5306A; 2.5 μg) for standards, and a rabbit anti-rat LH serum (NIDDK-anti-rLH-S-10) for use at a final dilution of 1:750,000. The detection limit of the assay was 0.01953 ng/tube, or 0.2 ng/ml. A mouse serum pool (ET-mouse #4) was used in triplicate in each assay as quality controls. The intra- and inter-assay coefficients of variation were less than 10%.

Corticosterone (blood) and ACTH in pituitary cell culture medium and in mice were measured by ether extraction and RIA at the Endocrine Technology and Support Core Lab (ETSL) at the Oregon National Primate Research Center/Oregon Health and Science University (Rasmussen et al., 1984). Briefly, samples for CS (1–3 μl) were extracted in 5 ml of ether in 13 mm × 100 mm glass tubes (baked at 500°C for 30 min), dried under forced air, and analyzed by specific CS RIA. Hormonal values were corrected for extraction losses determined by radioactive trace recovery at the same time with sample extraction; hot recovery was usually greater than 90%. Assay sensitivity was 5 pg/tube. The intra- and inter-assay coefficients of variation were less than 10% and 15% for cortisol and ACTH, respectively.

### Brain Slice Electrophysiology

#### Brain Slice Preparation

Slices containing GnRH neurons were obtained from GnRH-GFP transgenic mice, which express green fluorescent protein (GFP) in GnRH neurons (Spergel et al., 1999). On the day of the experiment, mice were anesthetized with isoflurane and decapitated. Brains were quickly removed and immersed in ice-cold high-sucrose solution containing (in mM) 220 sucrose, 2.5 KCl, 6 MgCl_2_, 1 CaCl_2_, 1.23 NaH_2_PO_4_, 26 NaHCO_3_, and 10 glucose (gassed with 95% O_2_/5% CO_2_; 300–305 mosmol l^-1^). Sagittal brain slices (300 μm thick) were prepared using a vibratome to cut slices containing the hypothalamus. Brain slices were transferred to an incubation chamber filled with artificial CSF (ACSF) solution containing (in mM) 124 NaCl, 3 KCl, 2 MgCl_2_, 2 CaCl_2_, 1.23 NaH_2_PO_4_, 26 NaHCO_3_, and 10 glucose (gassed with 95% O_2_/5% CO_2_; 300–305 mosmol l^-1^) and incubated at room temperature (22°C). After a 1–2 h recovery period, slices were transferred to a recording chamber mounted on an Axioskop microscope (Zeiss, Göttingen, Germany) and perfused with a continuous flow of gassed ACSF. Experiments were performed at room temperature.

#### Patch-Clamp Recording

Whole-cell patch-clamp recordings were performed on GFP-expressing neurons that were visualized using a GFP filter set and an infrared-differential interference contrast (DIC) optical system combined with a monochrome CCD camera and monitor. Pipettes used for whole-cell recording were pulled from thick-walled borosilicate glass capillary tubes (length 75 mm, outer diameter 2 mm, inner diameter 1 mm, Hilgenberg, Malsfeld, Germany) using a P-97 Flaming/Brown micropipette puller (Sutter Instrument, Novato, CA, United States) and had resistances ranging from 3 to 6 MΩ when filled with pipette solution, which contained (in mM) 145 potassium gluconate, 1 MgCl_2_, 10 HEPES, 1.1 EGTA, 2 Mg-ATP, 0.5 Na_2_-GTP, and 5 disodium phosphocreatine (pH 7.3 with KOH; 285–295 mosmol l^-1^). Pipettes were connected via an Ag–AgCl wire to the head stage of an EPC-9/2 patch-clamp amplifier (HEKA Instruments, Lambrecht, Germany). The reference electrode was a silver–silver chloride pellet (IVM, Healdsburg, CA, United States) immersed in the bath solution. Pipette and cell capacitance were compensated for using PatchMaster software (HEKA). The liquid junction potential was corrected for using the Blair method^1^. PatchMaster was used to acquire and analyze the data. Traces were processed for presentation using Igor Pro (Wavemetrics, Lake Oswego, OR, United States) and Canvas (ACD Systems International, Seattle, WA, United States) software.

#### Drugs and Drug Application

Corticotropin-releasing hormone (human, rat) was purchased from Tocris Bioscience (Bristol, United Kingdom). All other reagents were obtained from Sigma-Aldrich (Deisenhofen, Germany). CRH (and GABA, which was used as a control agonist) were prepared and stored as stock solutions according to the manufacturer’s instructions and diluted in ACSF to obtain the experimental concentration before each experiment. CRH (and GABA) were administered by a large diameter (300 μm) flow pipe, similar to the one used by Zhang and van den Pol (2013), with the tip directed toward the recorded cell. During periods of no drug application, normal ACSF was continuously supplied to the recorded cell through the flow pipe.

### Data and Statistical Analysis

Data are expressed as mean ± SEM. Differences between groups were examined using Student’s *t*-test, one-way, or two-way ANOVA followed by Bonferroni or Tukey’s multiple comparison tests. For analysis of the electrophysiological data, an unpaired *t*-test was used. All statistical analyses were performed with GraphPad Prism 7.0 software (GraphPad Scientific, San Diego, CA, United States). The level of statistical significance was set at *p* < 0.05.

### Histology

#### Double *in situ* Hybridization

For the simultaneous detection of CRHR1 mRNA and GnRH mRNA, six CRHR1GnRH-CKO mice treated with TAM, and six non-treated CRHR1GnRH-CKO mice were euthanized with CO_2_ inhalation. Brains were carefully removed and immediately frozen on dry ice, then stored at -80°C. Frozen brains were cryostat sectioned (Microm HM 560, Leica, Wetzlar, Germany) at 20 μm in the coronal plane and mounted on Superfrost Plus glass slides (Thermo Fisher Scientific). CRHR1 mRNA was detected with a radioactive labeled probe. GnRH mRNA was visualized with a digoxigenin (DIG) labeled probe. The riboprobes for CRHR1 mRNA and GnRH were previously published (Wray et al., 1989; Refojo et al., 2011). Utilizing PCR with T7 and T3 or SP6 primers, riboprobes were synthesized using plasmids containing cDNA from CRHR1 and GnRH as templates. Two hundred nanograms of the respective PCR product was used as a template to generate sense and antisense cRNA probes by *in vivo* transcription and to label them with DIG or with ^35^S-UTP. For the pretreatment on day 1, slides were thawed at room temperature for 30 min or until dry. Afterward, the pretreatment protocol was performed and slides were air-dried for 1 h. Hybridization was performed overnight with a concentration of 7 × 106 c.p.m. ml^-1^ for the radioactive CRHR1 probe and a concentration of 0.2 ng/μl for the non-radioactive GnRH probe at 56°C. The hybridization mix (hybmix: 50% formamide, 20 mM Tris-HCl pH 8, 300 mM NaCl, 5 mM EDTA pH 8, 10% dextran sulfate, 0.02% Ficoll 400, 0.02% polyvinylpyrrolidone 40, 0.02% BSA, 0.5 mg/ml tRNA, 0.2 mg/ml carrier DNA, 200 mM DTT) containing both probes was incubated at 92°C for 2 min, briefly put on ice, and then kept at room temperature. Ninety-five microliters of the hybmix were pipetted onto each slide. Slides were coverslipped and placed into a hybridization chamber overnight. On the following day, coverslips were removed from the slides and several washing steps were conducted. Subsequently, slides were incubated with Anti-DIG-POD 35 (Fab fragments) antibody (1:400; #11207733910, Roche) for DIG detection at 4°C overnight. Tyramide-biotin signal amplification (TSA; PerkinElmer, Waltham, MA, United States) was performed using the NEL700A Kit according to the manufacturer’s instructions, and was followed by streptavidin-AP (1:400) incubation for 15–30 min. VECTOR^®^ Red (Vector SK-5100; Vector Laboratories, Burlingame, CA, United States) was used for counterstaining. For radioactive CRHR1 *in situ* hybridization, slides were place into autoradiographic emulsion (type NTB2) and developed after 3–6 weeks.

### Histochemistry and Immunohistochemistry

Mice were euthanized with an overdose of Isofluran Baxter^®^ (Baxter, Unterschleissheim, Germany) and transcardially perfused with, 4% paraformaldehyde (PFA; Sigma-Aldrich). Free-floating vibratome brain sections (50 μm) were obtained. GnRHCreERt2 mouse lines were analyzed using X-gal staining combined with immunohistochemistry with antibodies against GnRH (rabbit, 1:3000; gift of Dr. Robert Benoit, McGill University). In parallel, sections were stained for Cre (rabbit, 1:1000; gift of Dr. Guenther Schuetz, DKFZ) and GnRH (rabbit, 1:1000; gift of Dr. Susan Wray, NIH). Sections from CRHR1-GFP mice were stained with antibodies against GnRH (rabbit, 1:3000; gift of Dr. Robert Benoit), GFP (chicken, 1:1000; Abcam, ab13970), and GAD67 (mouse, 1:2000; Millipore, Temecula, CA, United States, MAB 5406). Sections from CRHR2-Ai9-Cre mice were stained with an antibody against pre-pro GnRH (rabbit, 1:1:000; gift of Dr. Allan Herbison, University of Otago). Primary antibody-antigen complexes were detected using biotinylated antibodies (rabbit, 1:500, Vector Labs, Burlingame, CA, United States) and diaminobenzidine (DAB), or using fluorescent (CY3 or FITC-conjugated) antibodies (Jackson ImmunoResearch Labs, West Grove, PA, United States). Whole mount pituitaries of Nestin-Cre-Rosa mice were analyzed for β-galactosidase activity by the X-gal method. Pituitary sections from CRHR1- and CRHR2-Ai9-cre reporter mice were stained for LH (LHb, rabbit 1:1000, obtained from the National Hormone and Peptide Program, National Institutes of Diabetes, Digestive and Kidney Diseases, Bethesda, MD, United States, and Dr. Parlow, Harbor-UCLA Medical Center, Torrance, CA, United States; Chesnokova et al., 2007), and Alexa 488 anti-rabbit (1:1000) was used as secondary antibody (Life Technologies).

### Imaging

Diaminobenzidine-stained slice and dISH bright-field images were obtained with a Zeiss Axio Imager M1 inverted microscope (Carl Zeiss AG, Oberkochen, Germany) connected to a camera (AxioCam) controlled using AxioVision 4.8 software. Fluorescence analysis was performed using a Leica TCS SP2 and SP5 confocal laser scanning microscope (Leica) and Leica Laser Scanning System LSM510 software. To avoid channel crosstalk, FITC and Cy3 emissions were captured sequentially. Further image processing was performed with ImageJ 1.45J (NIMH, Bethesda, MD, United States), Fiji (NIMH), Adobe Photoshop CS3, or Illustrator CS3 (Adobe, Mountain View, CA, United States).

## Results

### Deletion of the Putative CRHR1 Gene in GnRH Neurons Does Not Prevent the Stress-Induced Decrease of Plasma LH Concentration

**FIGURE 2:**
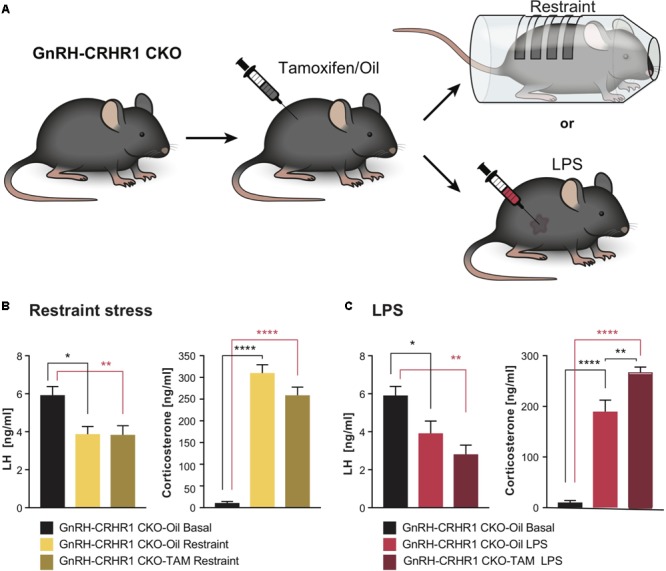
Experimental setups and plasma hormone concentrations in GnRH-CRHR1 CKO mice subjected to acute stressors. **(A)** Scheme of experimental setup: GnRH-CRHR1 CKO mice were treated with tamoxifen and 4 weeks later were subjected to a 1 h restraint and euthanized immediately after release from the restraint cylinder, or received an LPS injection (2 μg/g b.w., i.p.) and killed 1 h after the injection. **(B)** Restraint stress induced a significant decrease of plasma LH concentration in GnRH-CRHR1 CKO-Oil Restraint mice (*n* = 5) and in GnRH-CRHR1 CKO-TAM Restraint (*n* = 7) compared to basal concentrations in GnRH-CRHR1 CKO-Oil basal mice (*n* = 7). No difference was found between stressed GnRH-CRHR1 CKO mice, TAM and oil treated. One-way ANOVA, *F*(2, 16) = 7,56, *p* = 0.0049, followed by Tukey’s *post hoc* test: GnRH-CRHR1 CKO-Oil Basal vs. GnRH-CRHR1 CKO-Oil Restraint, ^∗^*p* = 0.0171, GnRH-CRHR1CKO-oil Basal vs. GnRH-CRHR1CKO-TAM Restraint, ^∗∗^*p* = 0.0081, and GnRH-CRHR1CKO-Oil Restraint vs. GnRH-CRHR1 CKO-TAM Restraint, NS, not significant, *p* = 0.9907. Restraint stress induced a significant increase of plasma CS concentration in GnRH-CRH-R1 CKO mice Oil and TAM treated compared to the basal levels of GnRH-CRHR1 CKO-Oil basal mice. One-way ANOVA, *F*(2, 16) = 136.5, *p* < 0.0001, followed by Tukey’s *post hoc* test (for GnRH-CRHR1 CKO-Oil Basal vs. GnRH-CRHR1 CKO-Oil Restraint and for GnRH-CRHR1 CKO-Oil Basal vs. GnRH-CRHR1 CKO-TAM Restraint, ^∗∗∗∗^*p* < 0.0001, and for GnRH-CRHR1 CKO-Oil Restraint vs. GnRH-CRHR1 CKO-TAM Restraint, NS, p = 0.0568). **(C)** LPS, like restraint stress, resulted in a similar decrease of plasma LH concentration in GnRH-CRHR1 CKO mice Oil-treated (*n* = 8) and TAM-treated (*n* = 9) compared to the basal plasma LH concentration of GnRH-CRHR1 CKO-Oil mice (*n* = 7). One-way ANOVA, *F*(2, 21) = 9.036, *p* = 0.0015, Tukey’s *post hoc* test: GnRH-CRHR1 CKO-Oil Basal vs. GnRH-CRHR1 CKO-Oil LPS, ^∗^*p* = 0.0387, GnRH-CRHR1 CKO-Oil Basal vs. GnRH-CRHR1 CKO-TAM LPS, ^∗∗^*p* = 0.0010, GnRH-CRHR1 CKO-Oil LPS vs. GnRH-CRHR1 CKO-TAM LPS, NS, not significant, *p* = 0.2786. For CS levels, ANOVA, *F*(2, 21) = 86.78, *p* < 0.0001, followed by Tukey’s multiple comparisons (GnRH-CRHR1 CKO-Oil Basal vs. GnRH-CRHR1 CKO-Oil LPS, ^∗∗∗∗^*p* < 0.0001, GnRH-CRHR1 CKO-Oil Basal vs. GnRH-CRHR1 CKO-TAM LPS, ^∗∗∗∗^*p* < 0.0001, and for the GnRH-CRHR1CKO-Oil LPS vs. GnRH-CRHR1 CKO-TAM LPS, ^∗∗^*p* = 0.0015.

In the course of our study, we used 14 lines/strains of transgenic mice, which are listed in **Table 1**. To ablate CRHR1 in GnRH neurons, we generated BAC transgenic mice expressing CreERt2 under the control of the GnRH promoter (**Figure 1**). After verifying the fidelity of Cre expression in GnRH neurons and the efficiency of Cre recombination induced by tamoxifen injection (see the section “*Materials and Methods”* and **Figure 1**), these mice were bred with CRHR1-2LoxP mice (Muller et al., 2003) to generate GnRH-CRHR1 conditional knockout (GnRH-CRHR1 CKO) mice. GnRH-CRHR1 CKO mice were treated with tamoxifen (1 mg, i.p., twice per day; see the section “*Materials and Methods”* for further details) for five consecutive days (Erdmann et al., 2007) and 3 weeks later were subjected to castration, which was followed a week later by stress experiments in which they were subjected to 60 min of restraint stress or received an injection of the bacterial endotoxin lipopolysaccharide (LPS, 2 μg/g b.w., i.p.; **Figure 2A**).

As depicted in **Figure 2B**, after 60 min of restraint stress, the LH levels of control (GnRH-CRHR1 CKO-Oil) and tamoxifen-treated (GnRH-CRHR1 CKO-TAM) mice were significantly reduced compared to the basal LH levels of the GnRH-CRHR1 CKO mice (*p* < 0.05: GnRH-CRHR1 CKO-Oil restraint vs. GnRH-CRHR1 CKO-Oil basal and *p* < 0.05: GnRH-CRHR1 CKO-TAM restraint vs. GnRH-CRHR1 CKO-Oil basal, respectively). However, the CS levels of both groups (GnRH-CRHR1 CKO_Oil restraint and GnRH-CRHR1 CKO-TAM restraint) were significantly higher (*p* < 0.001) than the basal CS levels of the control group (GnRH-CRHR1 CKO-Oil basal) (**Figure 2B**). One hour after the LPS injection (**Figure 2C**), the LH levels of GnRH-CRHR1 CKO-Oil and GnRH-CRHR1 CKO-TAM mice were significantly reduced compared to the basal LH levels of the GnRH-CRHR1 CKO mice (*p* < 0.05: GnRH-CRHR1 CKO-Oil LPS vs. GnRH-CRHR1 CKO-Oil basal and *p* < 0.001: GnRH-CRHR1 CKO-TAM LPS vs. GnRH-CRHR1 CKO-Oil basal, respectively). The CS levels of both groups (GnRH-CRHR1 CKO LPS and GnRH-CRHR1 CKO-TAM LPS) were significantly higher (*p* < 0.001) than those of the control group (GnRH-CRHR1 CKO-Oil) (**Figure 2C**). These results suggested that CRHR1s in GnRH neurons may not be involved in the stress-induced suppression of LH release.

### CRHR1 and CRHR2 Are Not Expressed in GnRH Neurons, and CRH Has No Effect on the Electrical Activity of GnRH Neurons

**FIGURE 3:**
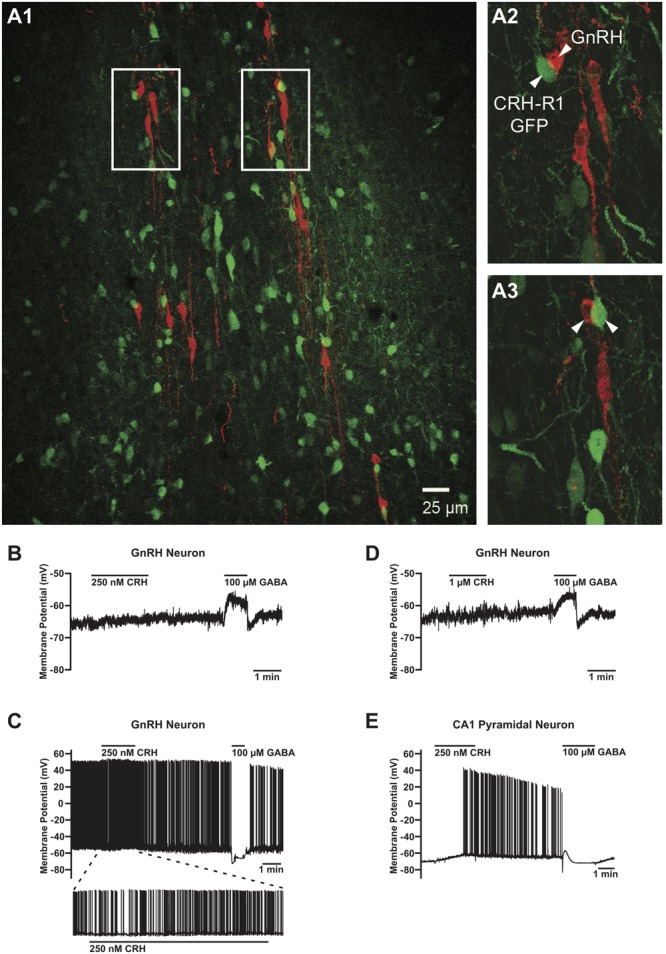
Absence of the CRHR1-GFP signal in GnRH neurons and lack of their electrophysiological responses to CRH. **(A)** GFP-positive neurons (green) in close proximity of GnRH neurons (red) **(A1)**. However, no GFP signals were found in any of the GnRH neurons analyzed. Arrowheads point to CRHR1-GFP and GnRH neurons. Scale bars = 100 μm **(A1)** and 10 μm **(A2, A3)**. **(B)** Lack of effect of 250 nM CRH on membrane potential in a non-firing GnRH neuron from a postnatal day (P) 44 GnRH-GFP mouse. **(C)** Lack of effect of 1 μM CRH on membrane potential in a non-firing GnRH neuron from a P36 GnRH-GFP mouse. **(D)** Lack of effect of 1 μM CRH on membrane potential and action potential firing in an active (i.e., spontaneously firing) GnRH neuron from a P38 GnRH-GFP mouse. **(E)** Depolarization and an increase in action potential firing evoked by 250 nM CRH in a non-firing hippocampal CA1 pyramidal neuron from a P37 GnRH-GFP mouse. GABA was applied after CRH to confirm the responsiveness of the cells to neurotransmitters/neuromodulators and the correct positioning of the drug application flow pipe. GnRH neurons responded to GABA with a depolarization **(B, D)** or a hyperpolarization **(C)**, and CA1 pyramidal neurons responded to GABA with a transient hyperpolarization followed by a transient depolarization and a sustained hyperpolarization **(E)**, in accordance with previous studies (Staley et al., 1995; Herbison and Moenter, 2011).

Since the presence of CRHR1 mRNA in GnRH neurons was previously reported in a single cell RT-PCR study (Jasoni et al., 2005), next we decided to verify the completeness of Cre-mediated deletion of CRHR1 using dISH, aiming to visualize CRHR1 mRNA in GnRH mRNA containing cell bodies of non-treated (oil) GnRH-CRHR1 CKO mice but not in those of TAM-treated GnRH-CRHR1 CKO mice. However, we were unable to detect specific CRHR1 mRNA signal in GnRH neurons of non-treated (oil) GnRH-CRHR1 CKO mice (**Supplementary Figure S2**) or, as expected, in GnRH neurons of GnRH-CRHR1 CKO mice treated with TAM (data not shown). To confirm these results, we employed brain sections of gonad-intact CRHR1-GFP BAC mice counterstained with GnRH antibodies. GFP signal (green) was detected in numerous neurons including some situated close to GnRH-immunoreactive neurons (red) (**Figures 3A1–A3**). However, no GFP signals were detected in cell bodies or processes of GnRH neurons in any of the 170 GnRH neurons analyzed (from three mice), indicating a lack of CRHR1 expression in GnRH neurons. Similarly, the analysis of brain sections of three gonad-intact CRHR2-Cre mice (Shemesh et al., 2016; Henckens et al., 2017) crossed with Ai9 mice carrying a floxed sequence of tdTomato revealed the absence of tdTomato in cell bodies or processes of GnRH neurons, indicating a lack of CRHR2 expression in GnRH neurons (**Supplementary Figure S4**).

We next performed an electrophysiological study with the local application of CRH onto GnRH neurons in the POA in sagittal brain slices of gonad-intact GnRH-GFP mice (**Table 1**). CRH (250 nM–1 μM) had negligible effects on membrane potential (**Figures 3B–D**) or action potential firing (**Figure 3C**) in GnRH neurons, as membrane potential in GnRH neurons changed by only 1.0 ± 0.3 mV (from -65.9 ± 2.9 mV to -65.0 ± 3.0 mV; *n* = 10 neurons from five mice) during 1–2 min of CRH treatment. In contrast, CRH (250 nM) markedly depolarized and excited hippocampal CA1 pyramidal neurons (**Figure 3E**). Membrane potential in CA1 pyramidal neurons changed by 4.9 ± 1.7 mV (from -67.9 ± 1.4 mV to -63.0 ± 3.0 mV; *n* = 3 neurons from two mice) during 1 min of CRH treatment, which was a significantly larger change than that in GnRH neurons (*p* = 0.003, unpaired *t*-test) and similar to previously reported responses of CA1 pyramidal neurons to 100–250 nM CRH (Kratzer et al., 2013). These results, which are consistent with the lack of CRHR1 expression in GnRH neurons (**Figure 3A**), suggest that CRH may act independently of GnRH neurons to decrease LH secretion. However, it remains possible that CRH acts indirectly on GnRH neurons, via presynaptic neurons, to decrease LH secretion but that CRH had no effect on GnRH neuron electrical activity in our experiments because connections between those presynaptic neurons and GnRH neurons were severed during slicing.

### Deletion of CRHR1 in Forebrain GABAergic Neurons or All Central Neurons and Glia Does Not Prevent Stress-Induced Inhibition of LH Release

**FIGURE 4:**
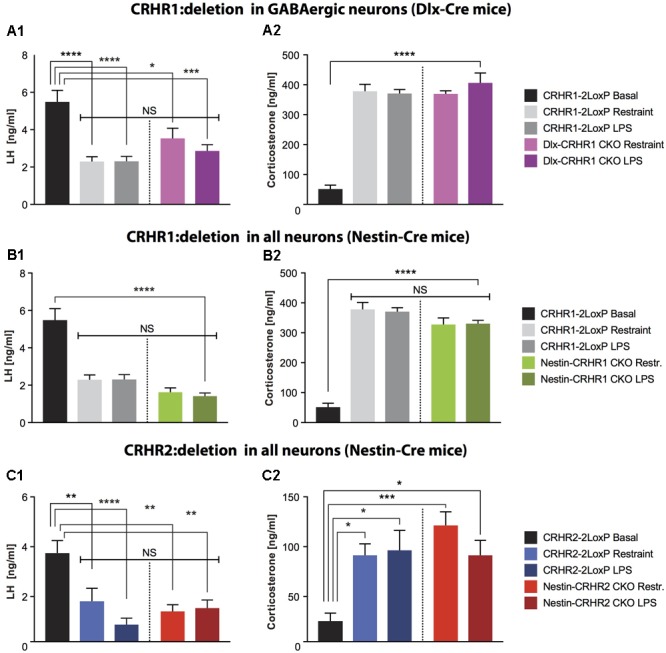
LH and CS concentrations in mice lacking CRHR1 in GABAergic neurons (Dlx-CRHR1 CKO mice) or in mice lacking CRHR1 or CRHR2 in all neurons (Nestin-Cre lines), subjected to restraint stress or LPS injection. **(A1)** Restraint stress (*n* = 5) and LPS (*n* = 8) decreased LH concentration in Dlx-CRHR1 CKO mice compared to non-stressed CRHR1-LoxP mice (*n* = 8). Stress-induced LH levels of control mice [CRHR1-2LoxP mice: Restraint (*n* = 12) and LPS (*n* = 12)] were similar to LH levels of the respective stressed Dlx-CRHR1 CKO mice, NS, *p* > 0.05 in all cases. One-way ANOVA, *F*(4, 40) = 14.23, *p* < 0.0001, followed by Tukey’s multiple comparisons test CRHR1-2LoxP Basal vs. CRHR1-2LoxP Restraint, ^∗∗∗∗^*p* < 0.0001, CRHR1-2LoxP Basal vs. CRHR1-2LoxP LPS, ^∗∗∗∗^*p* < 0.0001, CRHR1-2LoxP Basal vs. Dlx-CRHR1 CKO Restraint,^∗^*p* = 0.0174 and CRHR1-2LoxP Basal vs. Dlx-CRHR1 CKO LPS, ^∗∗∗^*p* = 0.0001. **(A2)** Restraint stress or LPS lead to equally increased CS levels in stressed animals of all groups. One-way ANOVA, *F*(4, 40) = 50.81, *p* < 0.0001, followed by Tukey’s multiple comparisons (CRHR1-2LoxP Basal vs. CRHR1-2LoxP Restraint, CRHR1-2LoxP Basal vs. CRHR1-2LoxP LPS, CRHR1-2LoxP Basal vs. Dlx-CRHR1 CKO Restraint and CRHR1-2LoxP Basal vs. Dlx-CRHR1 CKO LPS, ^∗∗∗∗^*p* < 0.0001 in all cases). **(B1)** Restraint stress and LPS induced a significant reduction in plasma LH levels in both controls (CRHR1-LoxP; Restraint, *n* = 12; LPS, *n* = 12) and Nestin-CRHR1 CKO mice (Restraint, *n* = 12; LPS, *n* = 12) compared to untreated CRHR1-2LoxP mice (*n* = 8). One-way ANOVA *F*(4, 48) = 28,38, *p* < 0.0001, followed by Tukey’s multiple comparisons test, CRHR1-2LoxP Basal vs. CRHR1-2LoxP Restraint, ^∗∗∗∗^*p* < 0.0001, CRHR1-2LoxP Basal vs. CRHR1-2LoxP LPS, ^∗∗∗∗^*p* < 0.0001, CRHR1-2LoxP Basal vs. Nestin-CRHR1 CKO Restraint, ^∗∗∗∗^*p* < 0.0001 and CRHR1-2LoxP Basal vs. Nestin-CRHR1 CKO LPS, ^∗∗∗∗^*p* < 0.0001. No significant differences were observed between the LH levels of stressed Nestin-CRHR1 CKO and those of stressed control mice (NS, *p* > 0.05 in all cases). **(B2)** Exposure to stress (either Restraint or LPS) resulted in a significant increase in plasma CS levels in both controls (CRHR1-2LoxP; Restraint, *n* = 12; LPS, *n* = 12) and Nestin-CRHR1 CKO mice (Restraint, *n* = 12; LPS, *n* = 12) compared to untreated CRHR1-2LoxP mice (*n* = 8). One-way ANOVA *F*(4, 48) = 53,75, *p* < 0.0001, followed by Tukey’s multiple comparisons tests CRHR1-2LoxP Basal vs. CRHR1-2LoxP Restraint, CRHR1-2LoxP Basal vs. CRHR1-2LoxP LPS, CRHR1-2LoxP Basal vs. Nestin-CRHR1 CKO Restraint, and CRHR1-2LoxP Basal vs. Nestin-CRHR1 CKO LPS, ^∗∗∗∗^*p* < 0.0001 in all cases. No significant differences were observed between the CS levels of stressed Nestin-CRHR1 CKO and those of stressed control mice (NS, *p* > 0.05 in all cases). **(C1)** Restraint stress and LPS led to a significant decrease in plasma LH levels in both controls (CRHR2-2LoxP; Restraint, *n* = 9; LPS, *n* = 9) and Nestin-CRHR2 CKO mice (Restraint, *n* = 9; LPS, *n* = 8) when compared to untreated CRHR2-2LoxP mice (*n* = 6). One-way ANOVA, *F*(4, 36) = 7.559, *p* = 0.0002, followed by Tukey’s multiple comparisons tests; CRHR2-2LoxP Basal vs. CRHR2-2LoxP Restraint, ^∗∗^*p* = 0.0088, CRHR2-2LoxP Basal vs. CRHR2-2LoxP LPS, ^∗∗∗∗^*p* < 0.0001, CRHR2-2LoxP Basal vs. Nestin-CRHR2 CKO Restraint ^∗∗^*p* = 0.0011, and CRHR2-2LoxP Basal vs. Nestin-CRHR2 CKO LPS, ^∗∗^*p* = 0.0030. No significant differences were observed between the LH levels of stressed Nestin-CRHR2 CKO and those of stressed control mice (NS, *p* > 0.05 in all cases). **(C2)** After restraint stress or LPS, the plasma concentration of CS was significantly increased in both control stressed mice (CRHR2-2LoxP; Restraint, *n* = 9; LPS, *n* = 9) and Nestin-CRHR2 CKO mice (Restraint, *n* = 9; LPS, *n* = 8) when compared to untreated CRHR2-2LoxP mice (*n* = 6). One-way ANOVA, *F*(4, 36) = 5.075, *p* = 0.0024, followed by Tukey’s multiple comparisons tests, CRHR2-2LoxP Basal vs. CRHR2-2LoxP Restraint, ^∗^*p* = 0.0326, CRHR2-2LoxP Basal vs. CRHR2-2LoxP LPS, ^∗^*p* = 0.0182, CRHR2-2LoxP Basal vs. Nestin-CRHR2 CKO Restraint ^∗∗∗^*p* = 0.0008 and CRHR2-2LoxP Basal vs. Nestin-CRHR2 CKO LPS, ^∗^*p* = 0.0388. No significant differences were observed between the CS levels of stressed Nestin-CRHR2 CKO and those of stressed control mice (NS, *p* > 0.05 in all cases).

Next, to determine whether GnRH neurons can be indirectly inhibited by GABAergic neurons during stress, we ablated CRHR1 in forebrain GABAergic neurons. GABAergic neurons provide a major input to GnRH neurons and may excite or inhibit them depending on the relative expression of GABA_A_ and GABA_B_ receptors, as well as the intracellular chloride concentration and resting membrane potential, of the GnRH neurons (Ondo, 1974; Herbison and Moenter, 2011). CRHR1 is expressed in different populations of GABAergic neurons (Refojo et al., 2011; Kuhne et al., 2012), potentially including neurons located near GnRH neurons, as we found in CRHR1-GFP mice (**Figure 3** and **Supplementary Figure S3**). To examine the functional role of CRHR1 in GABAergic neurons, we employed Dlx-CRHR1 CKO mice (**Table 1**), which were generated by crossing the well-characterized forebrain-specific Dlx5/6-Cre mice (Monory et al., 2006; Refojo et al., 2011) with CRHR1-LoxP mice (**Table 1**). As can be seen in **Figure 3**, LH concentrations were significantly decreased, while CS levels were significantly increased, in Dlx-CRHR1 CKO mice, similar to control groups subjected to restraint or LPS stress (**Figures 4A1,A2**). These results suggested that non-GABAergic neurons, in which CRHR1 was not deleted in Dlx-CRHR1 CKO mice, may contribute to stress-induced inhibition of LH release. To exclude the possibility that non-GABAergic neurons indirectly suppress GnRH neurons during acute stress, we employed Nestin-CRHR1 CKO mice (**Table 1**), which were generated by crossing Nestin-Cre mice with CRHR1-2LoxP mice and expressed Cre recombinase in all central neuronal and glial cells. CRHR1 expression in the brain has been shown to be completely abolished in the Nestin-CRHR1 CKO mice (Refojo et al., 2011). Similar to our findings with the Dlx-CRHR1 CKO mice, both stressors (restraint or LPS) suppressed LH concentrations in both Nestin-CRHR1 CKO and control mice (**Figure 4B1**), with a similar elevation of CS in both groups (**Figure 4B2**). These results suggested that CRHR1s in forebrain GABAergic neurons, or in any type of central neuron, or macroglia, may not be the only factors involved in the stress-induced suppression of LH release.

### Deletion of CRHR2 in All Central Neurons and Glia Does Not Prevent Stress-Induced Inhibition of LH Release

Since our results on deletion of putative CRHR1 in GnRH neurons, forebrain GABAergic neurons, and all central neurons and glia did not rescue stress-induced inhibition of LH release, we next ablated the second type of CRH receptor, CRHR2, which may contribute to CRH signaling despite having lower affinity than CRHR1 for CRH (Lovenberg et al., 1995; Grammatopoulos, 2012). After generating Nestin-CRHR2 CKO mice by breeding Nestin-Cre mice with CRHR2-2LoxP mice (**Table 1**), we subjected Nestin-HR2 CKO mice to acute stresses (either restraint or LPS) and found that, as with Nestin-CRHR1 CKO mice, LH concentrations were decreased to a similar extent as in the respective controls (**Figure 4C1**), while CS concentrations were elevated in both groups of stressed animals (**Figure 4C2**). These results suggested that CRHR2s in central neurons or glia may not be the only factors involved in the stress-induced suppression of LH release.

### CRH Infused Into the Brain Suppresses LH Concentrations via CRHR1 but Not CRHR2

**FIGURE 5:**
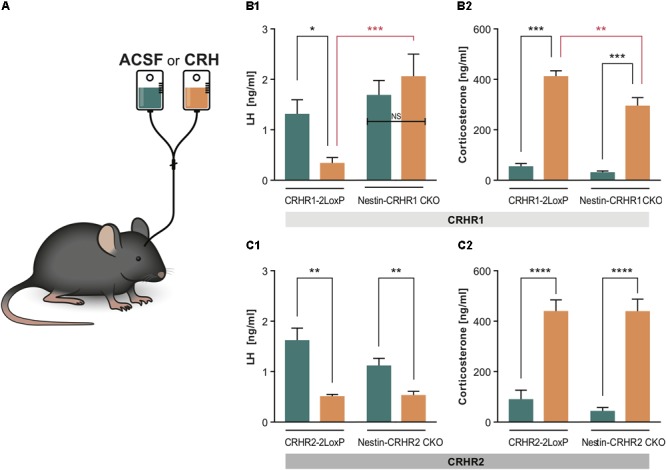
LH and CS concentrations in Nestin- CRHR1 and CRHR2 CKO mice after central CRH infusion. **(A)** The i.c.v. infusion of ACSF or CRH into the lateral ventricle. **(B1)** After i.c.v. CRH infusion, LH concentrations were significantly lower in control CRHR1-2LoxP mice (CRH, *n* = 8 vs. ACSF, *n* = 7), but not in Nestin-CRHR1 CKO mice (CRH, *n* = 7 vs. ACSF, *n* = 7). Two-way ANOVA, *F*(1, 25) = 13.75, *p* = 0.010, followed by Bonferroni’s multiple comparisons test (ACSF vs. CRH in CRHR1-2LoxP, ^∗^*p* = 0.043, and CRH vs. ACSF in Nestin-CRHR1 CKO, NS, *p* = 0.7539). Furthermore, after CRH infusion, the LH levels were significantly decreased in the control CHR1-2LoxP mice when compared to the LH levels of Nestin-CRHR1 CKO mice, ^∗∗∗^*p* = 0.0004. **(B2)** After CRH infusion, the CS levels were significantly increased in both groups of animals (either in CRHR1-2LoxP or Nestin-CRHR1 CKO mice) when compared to their respective CS levels after ASCF infusion. Two-way ANOVA, *F*(1, 25) = 6.67, *p* = 0.0160, followed by Bonferroni’s multiple comparisons test (CRH vs. ACSF in CRHR1-2LoxP mice, ^∗∗∗^*p* < 0.0001 and ACSF vs. CRH in Nestin-CRHR1 CKO mice, ^∗∗∗^*p* < 0.0001). Interestingly, the CS levels of the CKO mice were found to be statistically reduced when compared to those of CRHR1-2LoxP mice after CRH infusion (^∗∗^*p* < 0.05). **(C1)** In contrast, after CRH infusion, plasma LH concentrations were found to be significantly decreased in control CRHR2-2LoxP mice (ACSF *n* = 5 vs. CRH, *n* = 6), and in Nestin-CRHR2 CKO mice (CRH, *n* = 6 vs. ACSF, *n* = 5). Two-way ANOVA, *F*(1, 18) = 4.477, *p* = 0.0486, followed by Bonferroni’s multiple comparisons test (CRH vs. ACSF in CRHR2-2LoxP mice, ^∗∗^*p* < 0.001, and CRH vs. ACSF in Nestin-CRHR2 CKO mice, ^∗∗^*p* = 0.0069). **(C2)** After CRH infusion, the CS levels were significantly increased in both groups of animals (either in CRHR2-2LoxP or Nestin-CRHR2 CKO mice) when compared to their respective CS levels after ASCF infusion. Two-way ANOVA, *F*(1, 18) = 96,85, *p* < 0.0001, followed by Bonferroni’s multiple comparisons test (CRH vs. ACSF in CRHR2-2LoxP, ^∗∗∗∗^*p* < 0.0001, and CRH vs. ACSF in Nestin-CRHR2 CKO mice, ^∗∗∗∗^*p* < 0.0001).

Since all of the above results showed that deleting CRHR1 or CRHR2 did not reverse acute stress-induced inhibition of LH release, we infused 1 μl of human recombinant CRH [200 ng/1 μl; (Radulovic et al., 1999)] into the lateral ventricle of Nestin-CRHR1 CKO and Nestin-CRHR2 CKO mice (**Figure 5A** to determine whether CRH acts via CRHR1 or CRHR2 in the brain to reduce plasma LH concentrations. As depicted in **Figure 5B1**, CRH infusion decreased plasma LH concentrations in control mice (CRHR1-2LoxP mice) but not in Nestin-CRHR1 CKO mice. In contrast, after CRH infusion in Nestin-CRHR2 CKO mice, plasma LH concentrations were indistinguishable from those of control mice (**Figure 5C1**). However, the CS levels of CRHR1-2LoxP and Nestin-CRHR1 CKO mice infused with CRH were significantly higher compared to the CS levels of CRHR1-2LoxP and Nestin-CRHR1 CKO mice infused with ACSF (*p* < 0.001, **Figure 5B2**). Notably, after CRH infusion, the CS levels of Nestin-CRHR1 CKO mice were statistically lower than those of CRHR1-2LoxP mice (*p* < 0.05, **Figure 5B2**). In contrast, in Nestin-CRHR2 CKO mice we found a decrease of plasma LH concentrations after CRH infusion (**Figure 5C1**), while the concentrations of CS were equally elevated in Nestin-CRHR2 CKO mice and their respective control, CRHR2-2LoxP mice (**Figure 5C2**). These results suggest that CRHR1s in the brain mediate the suppression of LH release by exogenous CRH in non-stressed mice.

### CRH Applied to Pituitary Cell Cultures Suppresses LH Release via CRHR2 but Not CRHR1

**FIGURE 6:**
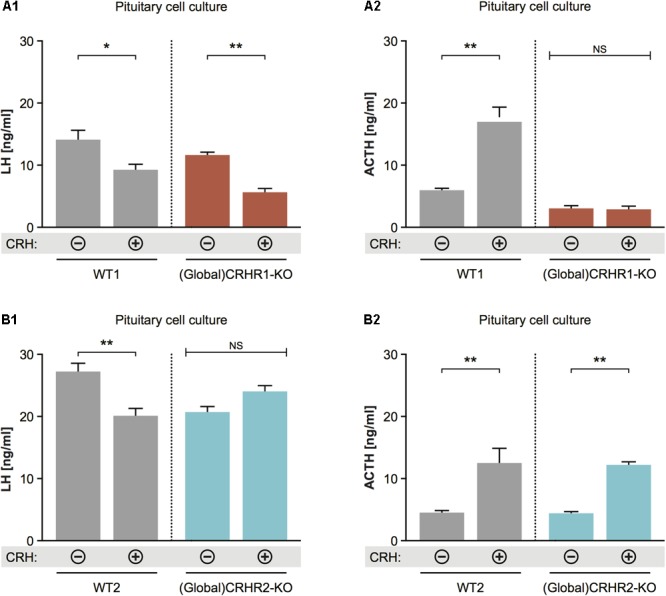
Effect of CRH on LH release from pituitaries of mice lacking CRHR1 or CRHR2. **(A1)** CRH application induced significant reduction in LH concentrations in the pituitary cultures of WT and CRHR1-KO mice. Two-way ANOVA, *F*(1, 8) = 9.412, *p* = 0.0154, followed by Bonferroni’s multiple comparisons test, CRH(+):WT1 vs. CRH(–):WT1, ^∗^*p* = 0.0187, and CRH(+):(Global)CRHR1-KO vs. CRH(–):(Global)CRHR1-KO, ^∗∗^*p* = 0.0044. **(A2)** ACTH concentrations were increased in the medium of pituitary cells from WT mice, but not from functional CRHR1-KO mice. Two-way ANOVA, *F*(1, 8) = 53.21, *p* < 0.0001, followed by Bonferroni’s multiple comparisons test, CRH(+):WT1 vs. CRH(–):WT1, ^∗∗∗^*p* = 0.003, whereas CRH(+):(Global)CRHR1-KO vs. CRH(–):(Global) CRHR1-KO, NS. **(B1)** CRH application induced significant reduction in LH concentrations in the culture medium of WT mice, but not in that of CRHR2-KO mice. Two-way ANOVA, *F*(1, 10) = 17.32, *p* = 0.0020, followed by Bonferroni’s multiple comparisons test, CRH(+):WT vs. CRH(–):WT2, ^∗∗^*p* = 0.0031 and CRH(+):(Global)CRHR2-KO vs. CRH(–):(Global)CRHR2-KO, NS, *p* > 0.05. **(B2)** ACTH levels in the medium of pituitary cell of WT littermates and global CRHR2-KO mice were equally increased after CRH stimulation in comparison to non-stimulated conditions. Two-way ANOVA, *F*(1, 10) = 34.75, *p* = 0.0002, followed by Bonferroni’s multiple comparisons test, CRH(+):WT vs. CRH(–):WT2, ^∗∗^*p* = 0.0021, and CRH(+):(Global)CRHR2-KO vs. CRH(–):(Global)CRHR2-KO, NS, *p* = 0.0065.

**FIGURE 7:**
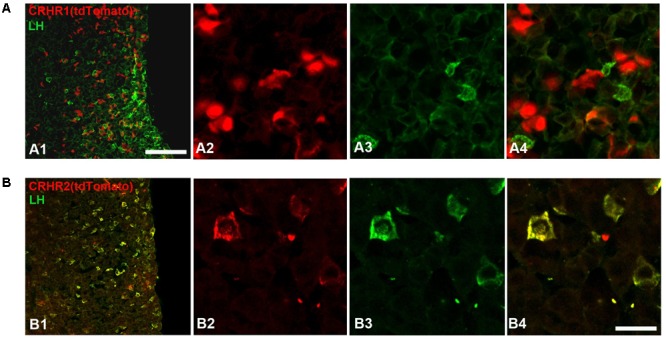
Expression of CRHRs in gonadotropes. **(A)** Representative pituitary section from a CRHR1-Cre/Ai9 mouse **(A1–A4)** stained with an antibody against LH (green). TdTomato signal was not found in LH cells. **(B)** Pituitary section from a CRHR2-Cre/Ai9 mouse **(B1,B2)** immunostained for LH. The cytoplasm and initial process of virtually all LH cells contain tdTomato (appearing as a yellow signal in **B4**). Scale bars = 500 μm **(A1,B1)** and 50 μm **(A2–A4, B2–B4)**.

To determine whether CRH can affect LH release at the pituitary level, and through which CRH receptor subtype it may act, we applied 10^-8^ M CRH (Blank et al., 1986; Smith et al., 1998; Henckens et al., 2017) to dissociated cell cultures prepared from the anterior pituitaries of CRHR1-KO (Refojo et al., 2011) and CRHR2-KO (Coste et al., 2000) mice and then measured LH concentrations in the culture medium. Two-way statistical analysis revealed that CRH application to pituitary cell cultures of wild-type (WT) mice decreased LH release and increased ACTH release into the culture medium (**Figures 6A1,A2**). In the cell cultures of pituitaries harvested from CRHR1-KO mice, CRH again decreased LH release; however, ACTH levels did not change significantly (**Figures 7A1,A2**). In contrast, ACTH levels in the culture medium of pituitaries harvested from CRHR2-KO mice were significantly elevated after CRH stimulation, but LH concentrations were unaffected (**Figures 6B1,B2**). These results suggest that CRHR2s in the pituitary, along with CRHR1s in the brain, mediate the suppression of LH release induced by exogenous CRH.

Finally, we analyzed the expression of CRHRs in pituitary gonadotropes (LH cells), employing CRHR1-Cre/Ai9 (Dedic et al., 2018) and CRHR2-Cre/Ai9 (Shemesh et al., 2016; Henckens et al., 2017) mice, which express tdTomato, under the control of CRHR1 or CRHR2 promoter-driven Cre recombinase, in the cytoplasm of cells having CRHR1 or CRHR2 receptors, respectively. In agreement with the results obtained in cell culture (**Figure 6A**), we were unable to detect tdTomato in LH-immunoreactive cells of CRHR1-Cre/Ai9 mice (*n* = 3; **Figure 7A**). In contrast, in CRHR2-Cre/Ai9 mice (*n* = 2) tdTomato signal was present in the vast majority of LH cells (**Figure 7B**). These results indicated that LH cells express CRHR2 but not CRHR1 and are consistent with our finding that CRH suppresses LH release in WT mice but not in CRHR2-KO mice (**Figure 6B**).

## Discussion

It is well known that stress negatively influences reproductive function. Yet, elucidating the signaling mechanisms through which stress interferes, at multiple levels, with reproductive axis activity, is an extremely complex research task due to the involvement of a variety of factors including species, sex and/or the levels of sex steroids, and the types of stressors (Chatterton, 1990; Tilbrook et al., 2000, 2002; Phumsatitpong and Moenter, 2018), which are known to have distinct and profound effects on the interaction of stress (HPA axis) and reproduction (HPG axis).

Besides the natural complexity that governs the relationship between stress and reproduction, there is a body of evidence (obtained in both human and animal studies) suggesting that the suppression of the HPG axis is at least partially due to elevated levels of endogenous CRH, the major stress hormone of the HPA axis, which is released in response to stress. For example, it has been shown that people suffering from a stress-related disorder, such as major depression, have increased levels of CRH and decreased reproductive function (Vermeulen, 1993; Gold and Chrousos, 2002). Furthermore, some studies report that CRH can exert a profound inhibitory effect on GnRH release and plasma LH levels (Ono et al., 1984; Rivier and Vale, 1984) in both *in vivo* and *in vitro* experiments (Gambacciani et al., 1986; Petraglia et al., 1987). Moreover, studies in rodents showed that an antagonist of CRH receptors prevents the suppression of LH release elicited by stressful stimuli (Rivier et al., 1986; Maeda et al., 1994). Interestingly, there are reports showing a direct synaptic connection between CRH neurons and GnRH neurons, at least in rats (MacLusky et al., 1988), as well as that a subpopulation of GnRH neurons in mice expresses CRHR1 (but not CRHR2) in brain areas including the MS, DBB, POA, and AHA (Jasoni et al., 2005; Todman et al., 2005).

Based on the above-mentioned hormonal, pharmacological, and anatomical evidence, the initial aim of the current study was to test the hypothesis that CRH acts via CRHR1 to inhibit reproductive function during acute stress. For this purpose, we generated a novel transgenic mouse line, GnRH-CreERt2, in which Cre recombinase is inducibly expressed in GnRH neurons under the control of the GnRH promoter and tamoxifen (**Figure 1**). After breeding this line with Rosa26 reporter mice, we used X-gal staining to confirm that Cre was expressed exclusively in GnRH neurons, which were identified using GnRH immunostaining, and as well as to monitor the efficiency of Cre recombination induced by tamoxifen (**Figures 1E1–E3**). This tamoxifen-inducible Cre system offers not only temporal control and cell-type specificity of Cre expression but also the benefit of being able to limit unwanted Cre activity as well as potentially toxic effects due to a prolonged Cre activation that have been reported in other Cre systems/lines (Schmidt et al., 2000). Taking advantage of this method, we generated the GnRH-CRHR1 CKO mouse line, in which we were able to delete CRHR1 in adult GnRH neurons. Surprisingly, our behavioral (stress) experiments and respective hormonal measurements using this mouse line showed that the absence of the putative CRHR1 in GnRH neurons did not prevent psychological (restraint) or immunological (LPS) stress-induced suppression of LH release. In support of our results and in order to investigate the potential direct effect of CRH on GnRH neurons through CRHR1s, we performed dISH for GnRH mRNA and CRHR1 mRNA in brain sections of GnRH-CRHR1 CKO mice. Interestingly, our data from the dISH could not confirm the presence of CRHR1 in GnRH neurons in the MS and POA (**Supplementary Figure S4**). These data are in agreement with those of Hahn et al. (2003), who also used dISH for CRHR1 mRNA and GnRH mRNA but were unable to detect CRHR1 mRNA in GnRH neurons of rats. It is also worth mentioning, that up to now, only one group has reported the presence of CRHR1 mRNA in GnRH neurons, by using DNA microarrays along with single cell reverse transcriptase (RT)-PCR and immunohistochemistry (Jasoni et al., 2005; Todman et al., 2005). It should be mentioned that their work was conducted using female mice while in our study male mice were used. Thus, it is possible that the discrepancy between our results and theirs is attributable to sex differences as well as to differences in techniques. In addition, the presence of CRHR1 mRNA does not necessarily indicate that the receptor is functionally active. Moreover, our electrophysiological data also support the idea that there is no direct effect of CRH on GnRH neurons since we showed that application of CRH did not change the electrical activity of GnRH neurons in male mice.

Our results are consistent with those of Jeong et al. (1999), who found that the global deletion of the CRH gene could not prevent stress-induced suppression of plasma LH levels, showing that plasma LH levels were decreased in both WT and CRH-KO mice following exposure to stressors (restraint or food withdrawal). Their finding raised the possibility that CRH alone may not be required for the suppression. In congruency, our results demonstrating that the deletion of CRHR1 in GnRH neurons or GABAergic neurons, or the deletion of either CRHR1 or CRHR2 in all central neurons and glia, could not prevent the suppression of LH release under acute stress (either restraint or LPS) suggest that CRH does not mediate the suppression (either by acting directly on GnRH neurons or indirectly through synaptic mechanisms) in the absence of central CRHRs. However, based on previous studies with CRH antagonists (Rivier et al., 1986; Maeda et al., 1994; Rivest and Rivier, 1995), it appears to at least partially mediate the suppression in the presence of central CRHRs (and may do so by acting indirectly on GnRH neurons, e.g., by inhibiting kisspeptin neurons; (Takumi et al., 2012; Grachev et al., 2013). Numerous studies have revealed a plethora of factors other than CRH involved in the suppression of reproductive function by stressful stimuli. For example, VP is co-expressed with CRH in the PVN upon chronic activation of the HPA axis (Plotsky, 1991; Aguilera, 1998; Grinevich et al., 2002), implying an involvement of this neuropeptide in stress. Several studies have demonstrated that VP can affect LH release: for example, infusion of VP into the lateral ventricles attenuates the preovulatory LH surge in rats (DePaolo et al., 1986) and causes a transient inhibition (although with lower potency than CRH) of LH release in male rats (Rivier and Vale, 1985). Extra-hypothalamic CRH acts rather as a neuromodulator than a neurotransmitter. However, it can be co-released with classical neurotransmitters, e.g., in the hippocampus with GABA (Yan et al., 1998) or in the locus coeruleus with GABA and glutamate (Valentino et al., 2001). Moreover, it has been shown that during stressful events, apart from vasopressin (Rivier and Vale, 1985; Heisler et al., 1994) which is co released with CRH, PACAP (Li et al., 1996), glucocorticoids (Breen and Mellon, 2014), urocortin 2 (Kageyama, 2013), interleukins (Rivest and Rivier, 1995), GnIH (Kirby et al., 2009; Ubuka et al., 2009), and many other factors act independently of CRH to suppress LH release, some of which may have compensated for the lack of CRHR1 or CRHR2 in the respective knockout mice in the present study. Thus, during stressful and life-threatening conditions, the inhibition of the HPG axis is ensured. This suppression is of the utmost importance for energy conservation and species survival (Barnea and Tal, 1991; Wasser, 1999). Additional studies are required to elucidate the mechanism whereby stress alters HPG function. Our findings provide a new impetus to search for factors besides CRH that are involved in the mechanism. An alternative explanation for our results is that CRH signaled through CRHR1 in the absence of CRHR2, and vice versa. Future studies employing CRHR1/CRHR2 double knockout mice are needed to address this possibility.

Interestingly, in our experiments the discrepancy between the effect of actual stress (induced by restraint or by injection of LPS) and of i.c.v. CRH infusion, which is widely used to partially mimic stress (Sutton et al., 1982; Weninger et al., 1999; Bryce and Floresco, 2016), on plasma LH levels lies partially within the above-mentioned framework. Thus, it does not seem so paradoxical that the deletion of CRHR1 in all central neurons and glia (Nestin-Cre line, see **Figure 5**) prevents the decrease in LH release following i.c.v. infusion of CRH but not the decrease in LH following actual stress (**Figure 4B1**). In our study, we used a lower dose of CRH (200 ng) than is usually applied [for example, 1 μg in (Gresack and Risbrough, 2011) and 1.5 μg in (Magalhaes et al., 2010)]. However, this dose was sufficient to induce stress responses, and this indicates that the discrepancy cannot be attributed to the dose of i.c.v. CRH infusion: as can be seen in **Figure 5B2**, i.c.v. infusion of CRH in control (CRHR1-2LoxP) mice induced an elevation of plasma CS concentration similar to that in mice subjected to actual stress (restraint and LPS; **Figure 4C2**). However, i.c.v. infusion of CRH in mice lacking CRHR1 in all central neurons and glia (Nestin-CRHR1 CKO) resulted in preserved plasma LH concentrations in contrast to control (CRHR1-2LoxP) mice, which exhibited a profound decrease in LH (**Figure 5B1**). Thus, the observed discrepancy may be attributable to differences between the effects of exogenously administered CRH and those of CRH released endogenously following stress. A somewhat analogous phenomenon has been reported for inhibitory effects of exogenous, but not endogenous, calcitonin gene-related peptide, on CRHR1-mediated pulsatile GnRH release during acute metabolic stress (Bowe et al., 2008). More importantly, apart from possible differences between CRH concentrations in brain extracellular fluid induced by i.c.v. infusion of CRH and those induced by stress (neither of which have been convincingly estimated so far by any lab), our work suggests that there are other factors (as mentioned above), such as vasopressin, cytokines, etc., which may inhibit LH release independently of CRH and CRHRs during actual stress.

**FIGURE 8:**
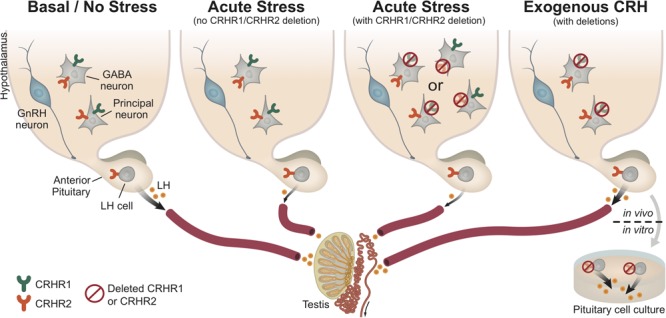
Central and pituitary CRH receptor signaling in the regulation of HPG axis activity. This schematic diagram summarizes our results and represents a working hypothesis regarding the distinct role of CRH receptors, at the pituitary and brain levels, under basal (non-stressful) and stressful conditions. As depicted in the first panel (from left to right), under basal conditions (no stress) the HPG axis demonstrates functional activity, which ultimately leads to normal LH release by gonadotropes (LH cells) in the pituitary. However, under acute stress (such as restraint stress or LPS injection; second panel), the HPG axis is disrupted (at all levels), resulting in reduced LH release. Interestingly, upon acute stress and deletion of either central CRHR1 or CRHR2 (third panel), but keeping CRHR1 and CRHR2 receptors at the pituitary level, we showed that the stress-induced LH release was also reduced. This finding indicates that upon stress the suppression of the HPG axis is not merely mediated through central CRHRs, but that other factors seem to be recruited and be responsible for the suppression of LH release. The last panel shows that CRH signals differently in the pituitary than in the brain: specifically, the deletion of CRHR2 (but not CRHR1) in the pituitary prevents the suppression of LH release from the pituitary (*in vitro*: pituitary cell cultures after stimulation with CRH), whereas the application of CRH (*in vivo*: i.c.v. infusion into the lateral ventricle) in unstressed mice lacking the CRHR1 (but not CRHR2) centrally also prevents the suppression of LH release. Both CRHR1 and CRHR2 are depicted in the same neurons for illustrative purposes.

Stress is known to impact the reproductive axis at multiple levels. However, the precise sites of action of CRH within the HPG axis (and particularly at the pituitary level) are not yet fully understood. In the present study, we also showed that CRH, in parallel with its central effects through CRHR1 (i.c.v. infusion of CRH, **Figure 5**), may act at the pituitary level through CRHR2 to suppress LH release (application of CRH to pituitary cell cultures of CRHR2-KO mice, **Figures 6, 8**). Although expression of both CRHR1 and CRHR2 has been reported in gonadotropes (Kageyama et al., 2003; Seasholtz et al., 2009; Westphal et al., 2009), our study, which employed transgenic mice, revealed that CRHR2, but not CRHR1, is expressed in native gonadotropes (**Figure 8**). It should be mentioned that the results of Seasholtz et al. (2009) and Westphal et al. (2009) were obtained using RT-PCR on an immortalized, cultured line of gonadotropes and therefore may not accurately reflect the expression of CRHR1 in native gonadotropes.

Studying HPA and HPG interactions in male mice undeniably offers a simpler model of reproductive function/physiology due to the lack of estrous cycle effects that are present in females. As females demonstrate a more complex interaction of the HPA and HPG axes than males (Novais et al., 2017), one limitation of our study lies in the fact that only male mice were used. However, any potential sex- or sex steroid-dependent differences were beyond the scope of the present study. Another limitation of our study is that only orchidectomized male mice were used for stress (and i.c.v. CRH infusion) experiments. However, without orchidectomy, plasma LH levels, already suppressed by the “negative feedback loop,” would have been further suppressed by stress treatments or i.c.v. CRH infusions, which would have made it difficult to detect LH or any potential differences in LH between experimental groups. Indeed, the pioneering works of Rivier et al. (1986) were based on castrated animals (male rats). Future studies that include gonadectomized as well as gonad-intact males and females, along with additional measures of reproductive function, are needed to cover this potential gap in the literature and to provide a more comprehensive picture.

## Conclusion

In conclusion, our results indicate that the deletion of CRH receptors does not prevent the inhibition of LH release following exposure to two entirely different types of stressor such as restraint and LPS stress. Additionally, our study suggests that CRH suppresses the basal activity of the HPG axis centrally via CRHR1 and at the pituitary level via CRHR2 (**Figure 8**). The impacts of these actions of CRH require further investigation, especially with regard to the mechanisms underlying human reproductive dysfunction associated with a modern lifestyle filled with stress (Gollenberg et al., 2010).

## Author Contributions

JD and VG conceived and designed the experiments. AR, LR, TB, CK, DS, and DG-G performed the experiments and analyzed the data. AR, DS, HM, JD, and VG extended manuscript preparation. AR, LR, DS, JD, and VG wrote the paper.

## Conflict of Interest Statement

The authors declare that the research was conducted in the absence of any commercial or financial relationships that could be construed as a potential conflict of interest.

## Footnotes

1 http://junctionpotential.blogspot.com/2010/10/how-do-i-measure-liquid-junction.html
