# Supplementary material for: Deciphering the Contributions of CRH Receptors in the Brain and Pituitary to Stress-Induced Inhibition of the Reproductive Axis
Source: Front Mol Neurosci. 2018 Aug 30;11:305. doi: 10.3389/fnmol.2018.00305 (PMC6125327; doi:10.3389/fnmol.2018.00305)
Supplement: Supplementary file 1 [file Data_Sheet_1.pdf]

SUPPLEMENTAL FIGURES

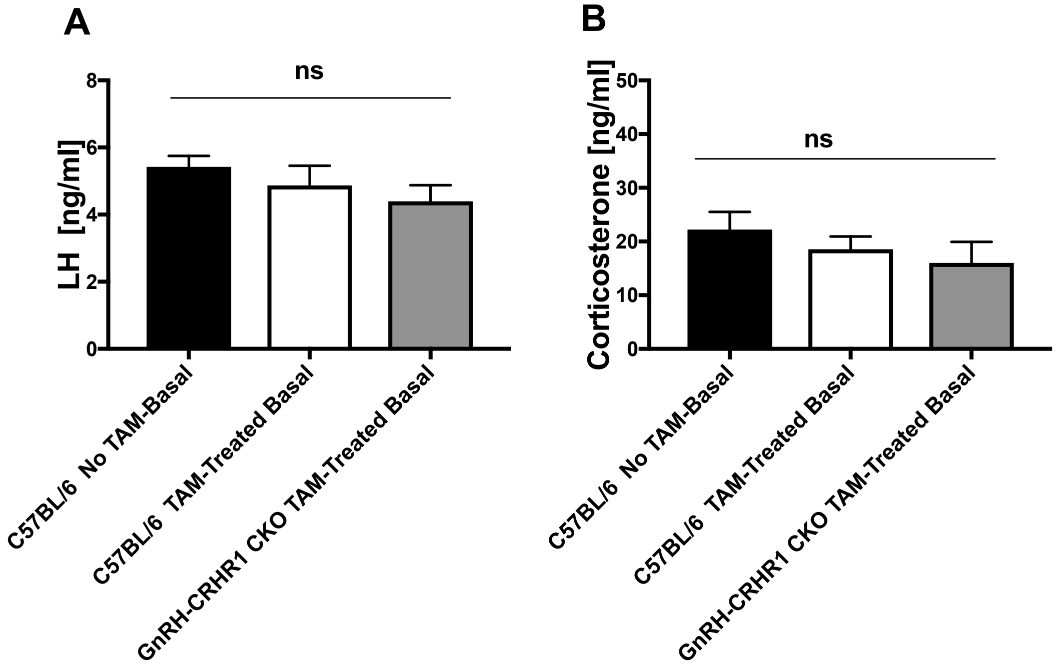

Figure S1. Plasma LH (**A**) and corticosterone (CS;**B**) levels under basal conditions in C57BL/6 (non TAM–treated) WT and in C57BL/6 WT and GnRH-CRHR1 CKO mice after five days of TAM injection (1 mg), twice a day. No statistically significant differences were observed among the three groups of animals. One-way ANOVA $F(2, 19) = 1.466$, $p=0.2558$ and $F(2, 19) = 0.7484$, $p=0.4866$ for the LH and CS levels, respectively.

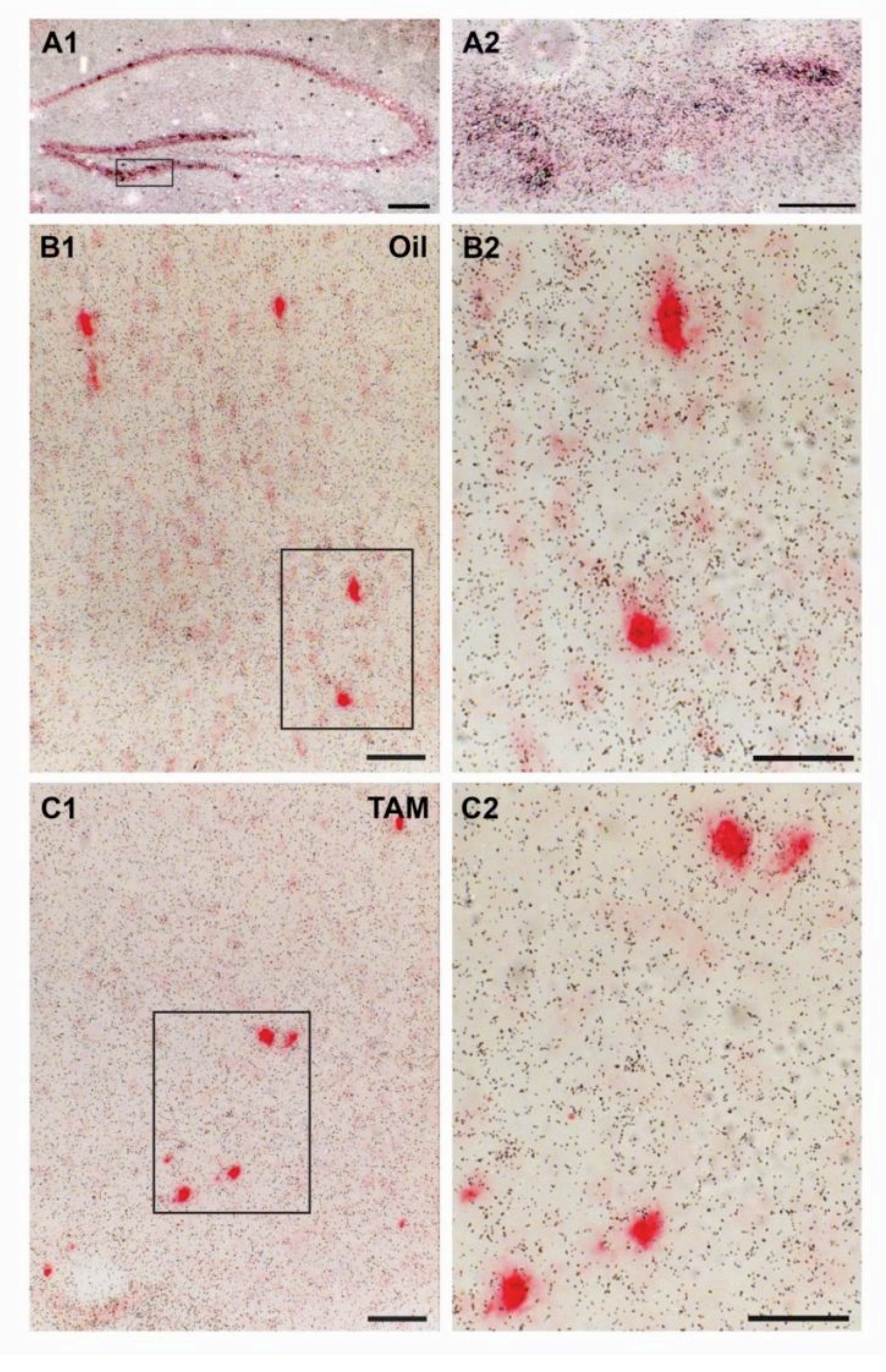

Figure S2. Lack of colocalization of CRHR1 and GnRH mRNAs, visualized using double radioactive- and DIG- based *in situ* hybridization.

A. *In situ* hybridization for CRHR1 mRNA (^{35}S-UTP-labeled probe; silver grains) resulted in a high density of silver grains above cells expressing CRHR1 mRNA in the hippocampus (A1, A2). Scale bars =

50 μm.

B. The number of silver grains above the cytoplasm of DIG-labeled GnRH neurons (red) in the medial septum of oil-treated GnRH-CRHR1 CKO (control; B1, B2) and tamoxifen-treated GnRH-CRHR1 CKO mice (C1, C2) was similar to that of the background. Scale bars = 50 μm.

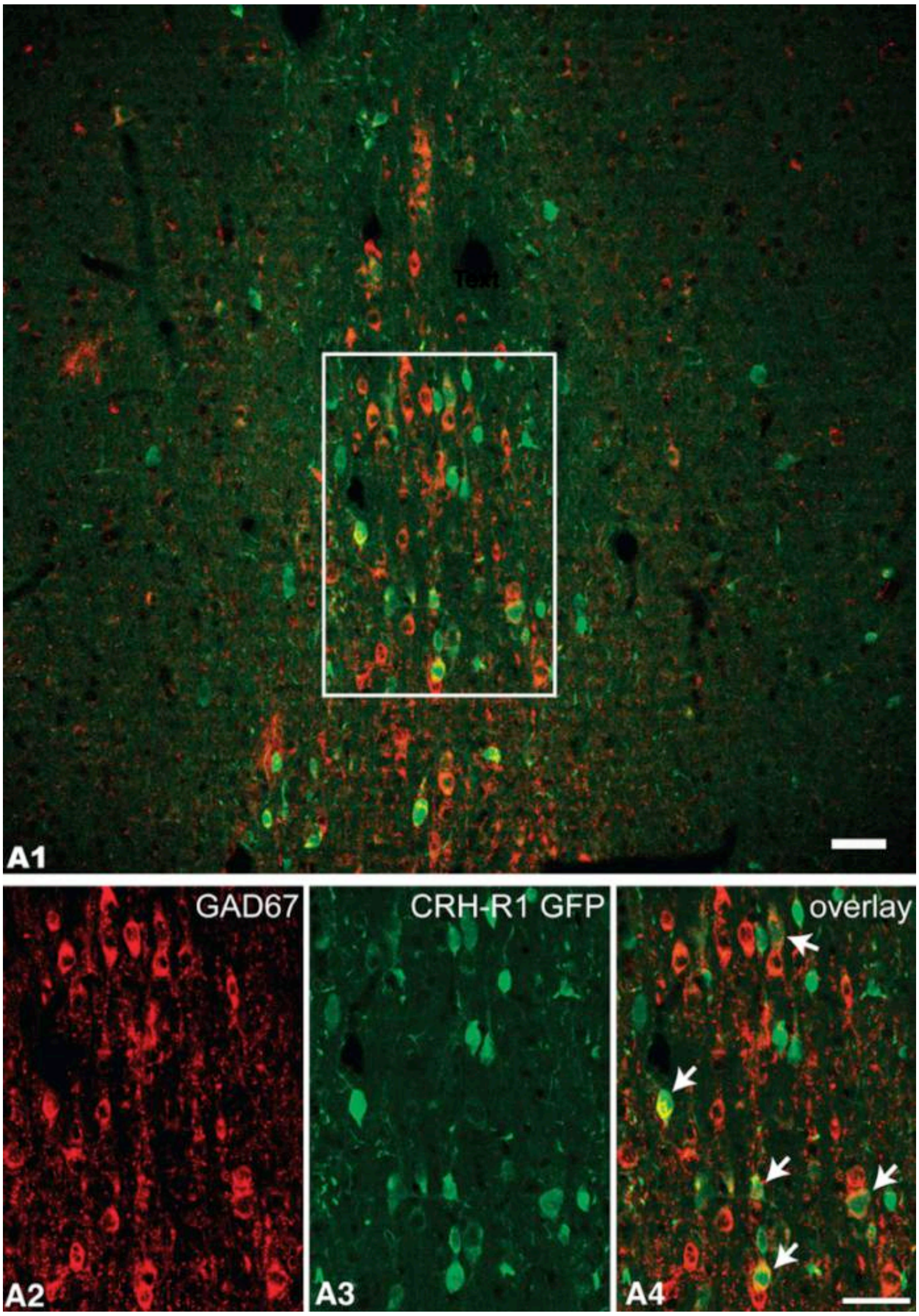

Figure S3. CRHR1-GFP signal overlaps with GAD67-immunopositive cell bodies in the preoptic area.

Staining of sections of the preoptic area of CRHR1-GFP BAC mice with a glutamic acid decarboxylase antibody (GAD67 red; **A1**) showed that GAD67 (**A2**) and GFP (**A3**) signals overlap in a fraction of GFP-positive neurons (yellow, **A4**), indicated with arrows. Scale bars = 50 µm.

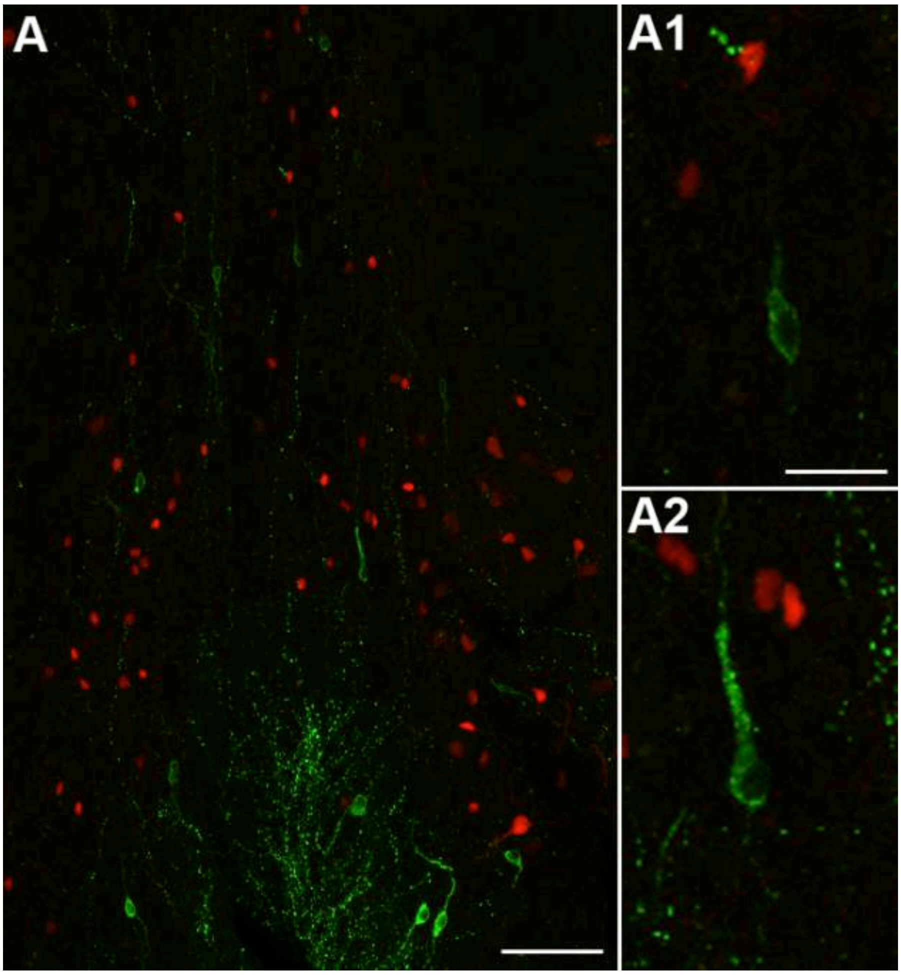

Figure S4. CRHR2-tdTomato signal is not present in GnRH neurons. No colocalization of tdTomato (red) and GnRH immunoreactivity (green) was found in cells located in the preoptic area at the level of the organum vasculosum of the lamina terminalis, as depicted at low (A) and high (A1 and A2) magnification. Scale bars = 100 µm (A) and 25 µm (A1, A2).
